# Lay persons’ perception of the requirements for research in emergency obstetric and newborn care

**DOI:** 10.1186/s12910-020-00568-1

**Published:** 2021-01-02

**Authors:** Dan Kabonge Kaye

**Affiliations:** 1 Department of Obstetrics and Gynecology, College of Health Sciences, Makerere University, P.O. Box 7072, Kampala, Uganda; 2 Berman Institute of Bioethics, Johns Hopkins University, Deering Hall, 1809 Ashland Avenue, Baltimore, MD 21205 USA

**Keywords:** Randomized clinical trials, Emergency obstetric and newborn care, Informed consent, Lay understanding, Clinical trial participation, Communitarianism, Solidarity

## Abstract

**Background:**

Factors that could potentially act as facilitators and barriers to successful recruitment strategies in perinatal clinical trials are not well documented. The objective was to assess lay persons’ understanding of the informed consent for randomized clinical trial in emergency obstetric and newborn care.

**Methods:**

This was a qualitative study conducted among survivors of severe obstetric complications who were attending the post-natal clinic of Kawempe National Referral Hospital, Uganda, 6–8 weeks after surviving severe obstetric complications during pregnancy or childbirth. The study that involved 18 in-depth interviews was conducted from June 1, 2019 to July 6, 2019. The issues explored included perceptions of the purpose and necessity to conduct such research how research-related information would be disclosed, and what could be the potential benefits and risks of participation. The data was analyzed by thematic analysis.

**Results:**

Respondents felt that research was necessary to investigate the cause, prevention or complications of an illness, especially as much was known about some pregnancy and newborn complications. Most believed that the emergency contexts affects whether and what prospective participants may understand if information about research was disclosed. Whereas they did not see the value of procedures like randomization, they felt that if these and any other procedures necessary should be done transparently and fairly. The decisions to participate would significantly be influenced by possibility of risk to the unborn baby or the newborn. Solidarity was an important influence on decision-making.

**Conclusions:**

Respondents valued participation in RCTs in emergency obstetric and newborn care. However, they expressed concerns and valued openness, transparency and accountability with regard to how clinical trials information is disclosed and the decision-making process for clinical trial participation. While autonomy and solidarity are contradictory values, they complement each other during decision-making for informed consent.

## Background

The expanded definition of a clinical trial is “…research study in which one or more human subjects are prospectively assigned to one or more interventions (which may include placebo or other control) to evaluate the effects of those interventions on health-related biomedical or behavioral outcomes.” [1]. In pregnancy and perinatal emergency contexts, obtaining informed consent for randomized clinical trials (RCTs) has potential practical and ethical challenges related to disclosure, comprehension and capacity (or competence) for voluntary authorization to participate) [2–7]. These challenges include inability to communicate to (and get consent from) very sick, anxious, unconscious or sedated patients, depending on how severe the patient’s condition and what medication they are taking or have already received at the time of the informed consent process. Some conditions in emergency obstetric and newborn care (such as eclampsia, abruptio placenta, postpartum hemorrhage, obstructed labor, birth asphyxia and neonatal sepsis) exist mainly as emergencies, and are the major causes of morbidity and mortality. Critically ill patients frequently undergo emergency treatment that affects their cognition (and therefore capacity to comprehend disclosed information about clinical trial participation) and subsequently provide voluntary informed consent. Yet consent for participation requires that potential participants understand the research purpose and procedures, the benefits and risks inherent in the RCTs, and the expected roles from them as research participants, before they give voluntary authorization to participate in research. The need and procedures for informed consent may need to be modified due to severity of the disease or the participants’ clinical state [2–7]. Potential participants may have incorrect interpretations of the requirements for informed consent [5, 6, 8].

Challenges in recruitment to RCTs hinder enrolment, continuation and successful completion of clinical research. For instance, problems with recruitment can also lead to low or selective enrolment, causing low sample size or selection bias respectively, thereby affecting the generalizability of trial results. Also, with insufficient numbers, RCTs may be underpowered to detect clinically meaningful differences in important outcomes in different study subject groups [9], substantially reducing validity and utility of the study findings [10–12]. However, informed consent for maternal and perinatal trials is often challenging as women and newborns are vulnerable at the time consent is required and mothers may have difficulty in making fully informed decisions [10–12]. What participants understand about the informed consent in emergency research is not well documented in emergency obstetric and newborn care. Some participants go through the process of informed consent when they have already made up their minds to accept research participation if invited [13], particularly in expectation of free medical care for them or their community [7, 14].

It is necessary to assess lay understanding of the informed consent process for RCTs in emergency obstetric and newborn care, as this may identify barriers and enablers to successful recruitment and strategies in perinatal clinical trials. What survivors of severe obstetric complications, who are potential participants of emergency research understand as requirements for emergency research acceptability is not documented. The objective was to assess and gain a deeper understanding of what lay people understand as requirements for acceptability of emergency research.

## Methods

### Data collection procedure

The study participants were 18 survivors of severe obstetric complications who were recruited for the study from the postnatal clinic of Kawempe National Referral Hospital, which they attended at least 6 weeks after childbirth. The study inclusion criteria were age of 16 years or older and a history of severe obstetric complication during pregnancy or childbirth. To verify eligibility, the patients’ medical records to confirm that they had suffered severe obstetric complications during their pregnancy or childbirth. Data were collected through in-depth interviews, using an interview guide developed and pretested specifically for this study. Since the objective was to explore participants’ perceptions and views related to research in emergency obstetric and newborn care, the issues explored included: (1) Perceptions of the value (necessity and relevance) and acceptability of conducting research in emergency obstetric and newborn care; (2) Perceptions on the procedures and processes for disclosure of clinical trial information (that is, how and when should potential participants be informed about the research and what factors may affect comprehension of disclosed information. Factors such as severity of disease, language, decision aids, different ways in which the information may be presented and contextual factors such as research environment, privacy, confidentiality were probed); (3) Participant perception of potential harms and risks that may arise from research participation. With prior permission from study participants, the interviews were audio recorded and field notes were taken to provide an opportunity to identify impressions, contexts, behaviors, and nonverbal communications, which could be relevant to how participants understand research and what meanings they attach to their decision-making to participate in research during emergency obstetric and newborn care. The interviews were conducted in either English or Luganda, a widely understood local dialect, which, though not necessarily all the participants’ mother tongue, is widely spoken and understood by many people in the capital city, Kampala. While all respondents understood both languages, the interviews and consent process were conducted in English for 12 participants, in Luganda only for 3 participants and both English and Luganda (3 for 3 participants).

### Data analysis

The NVIVO software was used for data analysis. Thematic analysis, as adapted from Morse [15] and Mays and Pope [16, 17] and based on the constant comparative method [18–20], was used to analyze for codes (recurrent patterns statements, words or phrases) with similar meaning or interpretation across data set [18–23]. During data analysis, audio recordings were transcribed verbatim by the investigator by listening to the audio recordings, transcribing the recordings and comparing them with the field notes. Field notes were checked for important contextual information that improved interpretation of audio-taped data. The transcripts were read carefully while listening to the audio recording so as to correct any areas that needed clarification, to correct spelling or other errors, and to anonymize the transcripts (so that the individual participants could not be identified from the transcripts (by deleting the names, places or significant events in the transcripts). From the field notes, information such as pauses, laughter, looks of discomfort and other contextual information collected were inserted into the transcripts. Once identified, the codes were aggregated into themes (groups of word patterns or phrases with similar meaning). Representative quotes from the individual transcripts were included to illustrate the source of the interpretations of the information from participants, and are presented in the results.

### Ethical considerations

This research was reviewed and approved by the Makerere University School of Medicine Research and Ethics Committee (SOMREC) and Mulago Hospital Research and Ethics Committee. The Institutional Review Board (IRB) of Johns Hopkins University School of Public Health relied on SOMREC as the IRB of record. Permission to conduct the study was also obtained from the Department of Obstetrics and Gynecology, Makerere University. While participation in this research was voluntary and respondents provided written informed consent, all prospective participants were given assurance that even if they declined to participate, their decision would not affect the due care that they were entitled to. Since the study posed potential risk of psychological distress if individuals were reminded of unpleasant past experiences during their prior hospitalization with acute illness, the information shared about unpleasant experiences was received in an empathetic manner. Participants who seemed emotionally affected by recall of unpleasant experiences during pregnancy or childbirth were given further counseling.

## Results

### Acceptability of research in emergency obstetric and newborn care

Many survivors of pregnancy complications had the view that investigations about the cause of illness was necessary, reasons given being that this was the way to develop new medications, to improve on existing medications, or to identify how to make better women who develop illness during pregnancy. The view that there was something new that needed to be understood was the major reason survivors found research in emergency obstetric and newborn care acceptable and relevant, as exemplified by two respondents:

Respondent 7: “There is always a lot that is not known about illness in pregnancy. And sometimes emergencies occur suddenly. On one day you are fine, the next day you are very sick. Even doctors cannot explain why the diseases occur… It is necessary to find answers to those questions.”

Respondent 2: “…finding out better ways of treating disease is a good (for conducting research or accepting to participate in research). Investigations provide answers to many questions about the disease… They are therefore necessary…even when you know that gathering the evidence requires many pregnant women to be involved…it may lead to some dangers of pregnant women, with all the risks that (participation) entails…but it is necessary”

However, especially when asked about whether it is acceptable for themselves or their newborns to be involved in those investigations, some respondents were of the view that in such situations, investigations were not the priority. Rather, it was providing the necessary emergency care that was a priority, but acceptance would depend on how the information was communicated, on whether the investigations were necessary, and whether the mothers understood the given information, as exemplified by two of the respondents.

Respondent 1: “There is often so much happening when someone is sick…when you come in with a sick baby. …this may occur when you have gone through a difficult pregnancy…why the hurry?… There is no need to waste time on being involved …You are worried about your baby…What you need then is treatment… Your health and that of the child take priority. …Taking part in other activities is the last thing on your mind.”

Respondent 2: “I wouldn’t accept to take part. You should not go beyond your limits. If are already sick or your child is in danger, there is no reason to add on to that danger…is it so important at that time? …unless there is hope of getting something better for you or your baby… I cannot even recommend what I do not support…###

On the timing of the research participation, some respondents felt that participation would be more acceptable if their concerns, like management of the emergency or newborn complications, are addressed, before enrolling them in clinical research. However, many women suggested that depending on how the information given or how persuasive the approach used was, they could accept to participate:

Respondent 18…Why would you take a risk if you don’t have to, or if there is nothing to gain? But if they tell you more about what is involved, so that they explain carefully, then (I) can accept.”

### Disclosure of information about research participation

On the timing of the research participation, some respondents felt that participation would be more acceptable if their concerns, like management of the emergency or newborn complications and the clinical condition has stabilized, as they were aware that in an emergency, participants may not clearly understand the disclosed information, may be unwilling to be delayed in the consenting procedures, or may be more likely to have therapeutic misconceptions. Regarding how invitation to participate should be communicated, most respondents were of the view that this should be when they, the unborn babies or their newborns were out of danger, and therefore when they could understand, without what was referred to as “pressure”, “stress” or “anxiety.” Some were of the view that if they received initial emergency care, then they had some obligation to participate in research as a way of showing their appreciation to services rendered and help already received, as exemplified by one respondent:

“…There is no need to waste time on being involved …What you need then is treatment… Your health and that of the child take priority. …Taking part in other activities is the last thing on your mind…But of course if you are helped and are better, you appreciate…You can accept to take part…Taking part shows you appreciate the help and are willing to help others in return…It is just natural…”.

The respondents preferred that the information would need to be simplified and brief, if they were to consider participating, as exemplified by two respondents:

Respondent 3: “You doctors have your difficult language… We don’t understand it. At that time what I need is help and care…I have no time to listen or understand those “heavy” medical terms. What they tell me should be short and simple, so that if I have to ask my friends for opinion, I would be able to tell them exactly what they (investigators) ask me to do.”

Respondent 5: “They should give me time. They may come and give me information, but should give me a written summary…something short…something to read on my own. If I can understand, then I may accept.”

Most respondents were of the view that they needed time to understand the information given about research participation, and that researchers should assess whether they have understood the basics about the study to confirm if they have understood, as demonstrated by two respondents:

Respondent 2: If you want me to take part, I may mess your study… You (have to) tell me about a new study that you want to do… You need to test me to check if I have understood. …Ask me a few questions about what I need to do and what you are going to do for me. If I answer correctly, then you guess that I have understood… If answers are wrong, then you repeat the information till I understand…like that…there should be no hurry.”

Respondent 5: “They should ask me, that okay, if you have understood, what is my role? …what do you want from me…What do are you going to do? What do you understand? Remember, at that time, your brain is not ‘steady’ and you may be tired. They need to check if you will do the right thing that they want. If it is taking the medicine correctly, they ask how many tablets, at what time, and what else you will be doing.”

Other respondents were of the view that the information about the research should be given privately, rather than to everyone present, and participation should be confidential, as exemplified by one respondent:

Respondent 5: “They need to come to you and then ask, are you going to take part?.. That should concern only me. I may not want other people, even my relatives to know that I am involved…that should be my secret…”

Most participants were of the view that participation may involve some degree of persuasion, and therefore those who recruit research participants should use a language the potential recruits are comfortable with and can easily understand. Besides, participation should involve possibility of personalized care in form of better attention to personal needs, as well as personal benefit for them, their unborn baby or the newborn. Regarding which potential personal benefits, the respondents suggested that they expect better care (including meeting the costs of investigations and medications, as exemplified by two respondents:

Respondent 1: “They have to convince me that it is a good idea. They need to talk to me in a language I understand best, not English… I expect that they would provide me with better care. For instance, get me the drugs I need, or give me better attention.”

Respondent 7: “At least they should contribute to some of my expenses like on drugs or food. They have to assure me that there is something to gain for me or my baby…I need to later be able to say, yes, taking part was a good idea… I want a higher standard of care if I participate. This can only be if I am better off than those who do not take part.”

On potential risks, the respondents were of the view that participation may lead one to spend a longer period of time in the health facility (because researchers need to continue with their study, or that some unexpected harm may occur. In case of possibility of significant harm, most respondents would either hesitate to participate, or would decline the invitation for participation, or may decide to discontinue participation, as exemplified by two respondents:

Respondent 8: “You never know what happens. If they give you medication that is not fully tested, the baby may be affected. Even me I may get complications. In that case I will have to decline or may leave their study.”

Respondent 6: “They may keep you longer in hospital because they still want you to do this or that procedure, taking off more blood, doing more checks on you… If what they tell me involves any of that (potentially harmful procedures), then I would not join… I would not want unnecessary delays.”

Regarding whether randomization, or the possibility of randomization to different groups may influence acceptability, most respondents indicated that they neither understood that procedure nor realized its importance or significance. Also, most were of the view that the investigators could decide what was best for them, and were ready to accept the procedure as long as they felt it was one way the investigators get the information they wanted from the studies. Therefore, they trusted the investigator to perform any procedure they deemed right, and would not mind getting involved in the randomization procedures, as exemplified by two respondents:

Respondent 7: “I do not understand why doctors would let me belong to one or other group just by chance. But they know better what they need to find out… I believe they would do the right thing… If what they do makes be belong to a particular group, I have no objection… I think they know better…I would not refuse.”

Respondent 5: “I think even when doctors say you should belong to this or that group, they know best… For me what would matter is that they do the right thing…they should ask me first… I believe they are called to serve. I have a feeling most doctors still do.

what is right for patients… Much as I do not understand how they would do it or why, I would believe that is the right thing (if they did it).”

### Solidarity was considered key in informed decision making

What was more apparent was that the potential participants may already have preconceived decisions about clinical trial participation or may rely on other individuals to influence their “informed” decision. This apparently impedes both voluntariness and autonomous decision-making, particularly in emergency situation where life may be at stake and decisions may need to be urgently made regarding clinical trial participation. These two possibilities may negatively or positively influence willingness and motivation for clinical trial participation in emergency obstetric care, as well as potentially influence discontinuation of clinical trial participation. This brings into question whether the decision for research participation in emergency obstetric care can be truly “informed” and autonomous, as one respondent shows:

Respondent 5: “Accepting research participations is not a simple decision…Community and friends advise or guide you in making a decision…You have to consult the spouse or your family doctor…This is the right thing to do…before you make the final decision.”

The respondents believed that the community played an integral role in the acceptability of the clinical trials (uptake, acceptability and integration of the clinical trial into the local setting). Patient decision-making whether or not to participate in the clinical trial was intimately tied to their relationship with others in their local community, whose advice was considered important for the decision whether or not to participate. However, inasmuch as respondents would seek advice on whether or not to participate, and would decide depending on what they considered important, they believed that individuals’ decisions were the most important. The respondents had expectations of mutual support in making such ‘important’ decisions. A number of participants reported that the benefits of the trial should be available to everyone involved, including other patients and community members, so because of that, their advice regarding participation was necessary. This would place the need for high standard of health care to be extended towards the patients in the investigation and that other community members would guarantee that the correct things are done. This is exemplified by two respondents:

Respondent 1: “I have ever looked after a mother who was involved in something like that. Before joining, we discussed the ideas and thought it would be a good thing. So we advised her to participate. At least even if there was not much good or benefit, there was nothing she would lose. So even for me, I would participate if invited, but I may need to consult my friends first.”

Respondents 3: “I would ask myself, is this a good thing for me? If I am convinced I would go ahead… This is an important decision. …If I have doubts, I would ask my friends or family members… Depending on their opinion, I would make my own decision (whether) to join (or not).”

Most respondents considered the advice of family members and friends as very crucial in decision-making, especially in emergency situations. While there was an apparent contradiction, the values of autonomy and solidarity seemed to complement each other:

Respondent 4: “It depends on how they convince me as to what I may benefit. Where I am not sure, I would ask for more time while I consult my friends or other patients. I may even call some nurses or family doctor. If they know better, they give advice…what they advise to do is what may help me to make the right decision… I think even if they were in my position, that is what they would do…If they asked me for advice, I would give it”.

The prior acceptance that such investigations should be conducted is a guarantee for easier acceptance of the invitation to participate, or likelihood of declining further participation or default by participants. Besides, the members in the community, not only those involved in the study, would share the blame rather than individual alone if something went wrong, and respondents justified the need to consult (or seek advice from others) as an obligation they owed to each other, more since even those who may not participate could potentially share from the research findings. This is exemplified by two respondents:

Respondent 3: “If the purpose if generating useful information, then it should not be a personal responsibility but a responsibility of everybody… This means that even the community members, the health facility managers and local political leaders should have a say about which investigations are acceptable… there should not be anything hidden…If they gave a green light prior, then that makes it easy for me to accept invitation to participate. Because then I know the major concerns that I may also have would have been addressed.”

The local political leaders and local influential people, such as doctors and nurses, have a significant influence on the trial acceptability. When the local leader is trusted by the community members and this individual approves of the trial, the potential study participants will be much more comfortable, as exemplified by other respondents:

Respondent 6: “ Our leaders ….consider what is important for us. …I believe they all have the best interests (of patients) on their mind as they deliberate about accepting such a study to be done.”

Respondent 9: “You can ask other nurses or doctors first for their opinion. If they are not sure about the study, you may decline immediately. If they are somehow positive, you may consider whether you can join. It all depending on how they approach you and what you think is important for you.”

Respondent 5: “If you have an opportunity to ask those already in the study, this can help you to make a decision. They can explain what is going an and what is involve. But you have to consider and make your own decision. If there is positive advice from your doctor or nurses, then there is no need for many questions.”

### Hope for some benefit as a driver for research participation

What is most striking in most of the interviews is the dominance of free medical care or some form of benefit as a motivation or predictor of willingness to participate in investigations under the prevailing circumstances. Limited capacity of local medical services to address emergency obstetric and newborn care needs is a major challenge in many health facilities in low and middle -income countries. Participation in research in a context where medical services in the clinical trial significantly surpass local services significantly increases risk of therapeutic misconception. Hope for some benefit is a key motivator for research participation, particularly for emergency cases, where high expenses may be involved during healthcare. The benefit may be material or therapeutic, suggesting that both therapeutic optimism and hope for material benefits are motivators for participation in research. This is exemplified by the following respondents. Therapeutic misconception in situations where research subjects fail to appreciate the distinctions between the imperatives of clinical research and ordinary clinical care, as components of clinical care occur in ongoing clinical research, thereby inaccurately attributing therapeutic intent to clinical research procedures including medication. Thus, such patients may overestimate the individual therapeutic potential of and value of their participation in clinical research. This wrong expectation of benefits may pose danger of therapeutic misconception and mis-estimation of risks.

Respondent 1: “I had to buy most of the drugs. If you are very sick they (doctors and nurses) tell you buy this and that medicine, that it will make you get better. Before the research study, when you visit the government hospital, you pay for the tests, you buy the medicines and sometimes you do not have the money. If they can provide the drugs I need, then I would take part. That may save me some of the expenses.”

Respondent 6: “ I am grateful that the nurses provided some drugs that I could not afford. I am grateful that both my child and I are okay now. Some friends lost their babies. Others die… If taking part solves the medical problem, then why not? I would not hesitate to participate … That may be my chance to help others.”

But other respondents suggested that participation would depend on how they are approached and how the invitation to participate was presented. However, a prior positive relationship cultivated with them by health workers would make it easier for them to make the decision to participate. This is exemplified by three respondents below:

Respondent 16: “It depends on how they approach me. If they are helpful with good manners, then I can accept. At times, you may go to the hospital and they ignore you, mistreat you or don’t help even when you call for help. Why should I join their studies? But If joining is my hope for getting help, I cannot hesitate to join…Some health workers are good, they are kind, they are supportive. If they could tell you to do something, you just accept because of their approach.”

Respondent 14: “Sometimes you hear rumors that those involved in research are given free drugs and they pay for the laboratory tests and other investigations. If they invited me and I knew they offered these, they I would not hesitate to join.”

### Compensation for dangers or risks of participation

On the issue of whether there should be compensation for adverse events or any harms that may arise during the research, most respondents were of the view that it is the responsibility of investigators to plan and provide compensation in case of any adverse events, whether foreseen or unforeseen. Participants were more concerned about harms or injuries that may cause death or disability (either serious or permanent). Respondents perceived that the responsibility of compensation should be guaranteed by the community or health facilities and should be enforceable. The compensation would be monetary, provision of treatment for the adverse event or both, but this would be dependent on the circumstances and different complications. If this did not happen, respondents felt that the researchers should be held responsible and could even be sued. This is exemplified by two respondents:

Respondent 1: “If the investigators suspect that my life may be put in danger, they should be ready for compensation by treating me or my baby, and even pay me some money for the trouble caused to me. …The society should guarantee that this happens …They should put the investigators to task if they fail.”

Respondent 7: “They find me with my illness and want me to get involved. They should compensate me (in case of problems). If not, they should leave me alone. They should not add to my trouble…They should take the blame…or else we end up in court.”

The most important concern was primarily whether there are risks for the baby, while the risks to the mother were secondary or not equally important. The main reasons given for giving the baby priority was that the baby needs to be able to grow optimally and survive the pregnancy, and may not survive if (research-related) complications arose. As mothers, most respondents would tolerate more risk than they would tolerate for the baby. However, most respondents were of the view that if research participation was to benefit the newborn or unborn baby, then they would not hesitate to join, as exemplified by several respondents:

Respondent 6: “As a mother I would accept quite a lot for that myself…Unless the risks are really dangerous… For my baby, there are a lot of things that I will not accept under all circumstances… However, if the danger is on my baby, then I will not hesitate.”

Respondent 8: “I have no reason to refuse. At least if they assure me that what the study involves will not hurt my baby… They have to convince me of benefits (for me or my baby). The information they tell me should be clear to me…Whatever procedures they will do (should be) safe for me and my baby… If am convinced, I can then be involved…If that is the only way to get help, I go for it”.

Respondent 4:“There is never an acceptable risk for my pregnancy. Never. I cannot accept to participate just to satisfy someone’s interests. A pregnant woman and her baby should be very much protected in our society… After all, a pregnancy is (like) an illness itself…I can understand that that the results may help me or others. But my interests and my concerns are more important.”

### Altruistic reasons versus solidarity

Pregnant women may participate in research for different reasons, for example because of altruistic or personal motives that potentially benefit others. Women had no problem in participating in research that would not include potential gain for themselves or the foetus, if this was mainly for other pregnant women or their unborn babies. They were also willing to participate in research that would only pose some risks to themselves and not to their foetus, if other women were to benefit. They suggested that the acceptable risk would be that which does not put them in any immediate or later danger or could make them worse (or feel worse) than they were at the time of invitation to participate in research. These motivations are demonstrated by the respondents below:

Respondent 6: (who had antepartum eclampsia): “May be for myself I may accept to participate even if I may not benefit, as long as my participation could benefit other women so that they do not suffer like me… Where my baby is involved, I cannot do anything like that”.

For research that involved invasive research procedures, respondents felt that they needed guarantees that these procedures should have a clear medical indication, as perceived by two respondents:

Respondent 1: “I am sure may women suffer the same problem I had. If it may put my baby in danger, I cannot accept anything like that. I would not accept anything that they are just trying out…May be some risk for me is acceptable, but for my baby, the danger should be zero if I am to accept for the sake of benefitting others. But I think many would also do the same for me”.

Respondent 6: “When I remember what I went through, I think it is important to help others in that critical period to also have this joy, to be able to appreciate my happiness that I went through this period well…There may be some danger. But if there is hope, then I would accept and go through it… May be this is God’s way of using me to help others… But I would leave immediately if I suspect some serious danger’.

## Discussion

There is limited research on lay persons’ understanding of clinical research, especially the requirements for informed consent. This limits ability to what factors could potentially motivate individuals to take part in RCTs in emergency obstetric and newborn care., and whether there are specific issues that clinician investigators need to be aware of or which they need to address to improve recruitment and retention into RCTs. Some of the issues relate to the approach used to recruit study participants, the information given to prospective participants, the methods used to communicate the information, the readability of the informed consent documents and the process used in seeking informed consent. Other features relate to the study design (choice of study procedures that reduce potential risks to participants, study benefits for participants, approach to data collection and compensation. Yet others relate to how the study is conducted, with openness, fairness and transparency. The study findings suggest that while autonomy and solidarity may appear as contradictory values, they complement each other.

The study findings show that the perceived level of risk to the unborn baby or newborn, much more than perceived potential risk to the mother, influences willingness to clinical trial participation. Secondly, hope of some benefit, either therapeutic or material, influences decisions for clinical trial participation. Thirdly, community solidarity and communal decision-making influence individual decisions, and may impede voluntariness and autonomous decision making for trial participation. Lastly, contextual factors, particularly poor access to needed health services and trust in the healthcare system influence willingness for clinical trial participation. Also, an opportunity for free medical care or assistance with aspects of medical care were strong motivators for clinical trial participation. In designing clinical trial protocols, investigators could aim at reducing potential factors that deter clinical trial participation [24].

The findings suggest that factors that may improve clinical trial enrolment include developing research questions which address issues that are relevant to the population (pregnant women, unborn babies or newborns) in the context in which the clinical trial is to be conducted [24, 25]. The findings demonstrate the interplay of personal, family, community and societal factors in influencing individual motivation to participate in clinical trial, in line with the socio-ecological framework. Personal-level factors include severity of illness, personal anxiety and fears about potential harms and risks of trial participation and possibility of personal benefit, either therapeutic or material from research participation. Family-level factors include the influence of friends and family members, either positively or negatively affecting individual decisions. Community level factors include challenges if the healthcare systems characterized by scarcities and high individual expenditure on health. The societal factors include expectations of solidarity and communal consent for participation in clinical trials of emergency obstetric care. Our findings are in agreement with other studies influence clinical trial acceptability and retention [25, 26]. The factors suggested by respondents show the different levels of influence where factors reinforce each other to affect an individual’s preference and values and eventually decision-making and choices. The findings indicate significant external influence of solidarity as reported in prior studies [27, 28].

The findings suggest that therapeutic optimism, therapeutic misconception (TM) and therapeutic misestimation may be common in emergency research during emergency obstetric and newborn care. TM is described as conflation of research with therapy. Patient-subjects manifest TM when they express an unreasonable or unrealistic appraisal of the nature or likelihood of medical benefit because of a misperception of the nature of the clinical research. Due to TM research subjects may be confused about the medical benefits (and risks) of participating in a clinical research setting or design per se. Participants may also manifest TM when they hold a false belief that their individual needs will determine assignment to treatment conditions or lead to modification of the treatment regime. Thus, they may conceal or misestimate the potential risks of research participation, especially if they hold high hopes of therapeutic benefit (therapeutic optimism). Also, TM represents failure by potential research participants to recognize limitations on individualized care and treatment that are inherent in certain clinical research methods and procedures because they do not operate on the principle of personal care.

Wherever possible, the trial design could use standard care as the basic treatment model [24, 25], so as to reduce potential participants therapeutic misconception and therapeutic misestimation optimism. TM has several implications for clinical research. Research participants may harbor TM if they misestimate the therapeutic potential or benefits of participating in trials with particular objectives and designs, such as early phase clinical research. Secondly, TM can even extend to surrogate decision-makers, such as spouses and relatives, who may be the persons available to give consent for participation in emergency research in situations where the patient is not in position to do so. Thus, parents who enroll their sick children in pediatric clinical trials may have TM secondary to holding high hopes or expectations of medical benefit for their children as an outcome of the research project, when in fact one or both of the medications under investigation may be experimental. Thirdly, TM may lead to misunderstanding that, in clinical research, it is possible that the investigational medication or procedure may not be equivalent to standard therapy, or may lead to undue optimism for the experimental intervention’s or medication because it is new, and therefore poses better prospects of medical benefit for patient-subjects. In addition, such TM may lead to unreasonable or unrealistic expectations of benefit. Fourthly, high hopes may lead them to underestimate the potential risk posed by the study medications or study procedures, thus the participants safety during research participation.

Therapeutic misconception and misestimation may exist together or independently. A research subject can fully understand the nature or intent of a clinical trial, when presented with clinical trial information during the informed consent process, but still misestimate the probability of medical benefits or risks. Conversely, a patient-subject can have realistic or reasonable expectations of therapeutic benefits and risks while conflating the nature of clinical research with the nature of clinical therapy. While therapeutic misestimation may be seen as less problematic than TM because it is a misrepresentation of an aspect (such as the probability of benefit and/or risk) of the option of research participation rather than a misrepresentation of the option itself, it curtails decision making regarding trial participation if the misestimation of benefit and/or risk is very large.

The findings suggest some potential factors that have to be incorporated into the participant recruitment plans for RCTs in emergency obstetric and newborn care. These include the types of information to be conveyed to potential participants, the formats used for that information (such as brochures, leaflets, radio and social media messages), the timing of requests for participation and for obtaining consent, and the approaches that may be used to enhance recruitment, such as community consultations. These impact significantly on women's and families' decisions to participate in perinatal RCTs [29–36]. A participant recruitment process that is aligned with potential participants’ views and values and fits into the standard clinical practices of health facilities is likely to result into higher rates of acceptance and retention for trial participation.

A carefully developed and streamlined recruitment process may also reduce tensions associated with trial participation [34–36].

The findings suggest that trust is also a key motivator for clinical trial participation [37] and is key to how individuals process the disclosed information, particularly the balance of risks and benefits of clinical trial participation. The provision of health care within the community context characterized by deficiencies influences trust in the investigators specifically and the healthcare system in general, further challenging both the motivation to participate in the clinical trial and the informed consent process [37]. Solidarity is another influence for research participation. The respondents were able to distinguish the ‘we-thinking’ of solidarity from the ‘other-thinking’ associated with altruism and charity. In altruism, there is concern for the wellbeing of others, without both an obligation to care for others and receiving (or even expectations of receiving) anything in return. Solidarity is thus born out of goodwill and awakens the virtue of goodwill among people [38], just as altruism, but this cannot be done at the expense of an individual’s risk. With solidarity, individuals who give out something are entitled to expectations of receiving something back, or reciprocity, as an indication of shared interests or characteristics. In the context of this paper, solidarity involves participation in research where individuals support (and have expectations of support for) each other in decision-making about research participation, yet individuals retain the autonomous choice of making the final decision. Also, solidarity is suggested by participation in research for the common good rather than individual good, and with hope or expectation of communal sharing of the research benefits [39]. The “common good” refers to those facilities, including material, cultural or institutional, that community members provide to all members in order to fulfill a relational obligation they all have to care for certain shared interests [40, 41].

However, solidarity obligations notwithstanding, individuals retain the moral agency to make the final decisions, indicating the complementary role of autonomy and solidarity. In this complementary role, some individuals may have the roles of gate-keepers or influencers, who facilitate or deter access to research participation by influencing choices, values and decision-making. This necessitates building openness, trust, trustworthy relationships, transparency and accountability between investigators and research participants and the community at large. Some argue that solidarity cannot achieve goals of social responsibility [42], others argue that harnessing the power of solidarity through community and stakeholder engagement achieves several goals, including promotion of efforts to protect host communities, promotion of initiatives for respecting host communities, promotion of transparency, and enhancement of the social value of the research [42, 43]. In any community, the common good refers to all those facilities and interests that members have a special obligation to care about considering that they exist in a certain relationship with one another.

Engaging communities can also have intrinsic and instrumental value beyond consent, as it may demonstrate respect for communities or identify appropriate ways of participant recruitment and informed consent. The consent process may be influenced or supported by the community and stakeholder engagement activities, which involves building authentic partnerships, mutual respect and active, inclusive participation, power sharing and equity. Engagement is important as community values, beliefs and norms tend to influence views on risks and benefits of research, thus affecting independent decision making or the consent process [43]. Thus, community and stakeholder engagement may provide insight on how to develop consent processes that are appropriate for the local context. Community and stakeholder engagement activities may also facilitate interpersonal communication which is vital for nurturing respect, comprehension of the purpose, risks and benefits of the research, explaining the need for and form of ancillary care obligations and compensation for research participation, and appreciating the community’s and participants’ contributions with post-trial benefits [44–46]. Community and stakeholder engagement is vital as there is interdependency between the influences on how people view risks and benefits of research, and how individuals value the research enterprises, thus affecting independent decision making or the consenting process [43–46].

Trust represents the power physicians have over their patients, yet such trust can be problematic in that it may be used (unconsciously or otherwise) to persuade or manipulate. In the emergency research context, certain researcher behaviors can persuade, manipulate, or coerce potential research subjects. Whether or not this trust (or power) creates positive or negative (undue) influence may be difficult to determine without consideration of the contextual factors and participant characteristics in emergency research. The concept of the risk of the controlling influence of research practices with the vulnerability of potential subjects shows that subjects are vulnerable because they are more likely to be unduly influenced (or unduly controlled) by any particular act of the researcher, and this act may include excessive trust. Manipulation may thus occur through manipulating options, manipulating information, or psychological manipulation, especially where there is high trust. In the emergency context, researchers may willingly fail to disclose all available information, especially withhold information about all the options and alternatives available in the clinical trial. Trust may also deter potential participants from demanding more information or indicating that they cannot comprehend the disclosed information.

## Conclusions

The respondents valued participation in RCTs in emergency obstetric and newborn care. However, they expressed perceptions with concerns on the way clinical trials information is disclosed, whether the information is understood, whether there is appropriate decision-making and existence of situations where participation may not be voluntary. Openness, transparency and accountability, especially in relation to potential benefits, potential research-related risks and compensation were perceived as critical requirements for an informed consent process. Investigators should ensure appropriate communication of clinical trial information to improve comprehension. They should also encourage shared decision-making, recognizing and taking into consideration prospective participants’ views and values. These values include therapeutic optimism and altruistic reasons. But they should also consider the potential role of therapeutic misconceptions and misestimation, have the potential to negatively influence the validity of the informed consent process.

The respondents perceived solidarity and communal decision-making as important in decisions to participate in emergency research, which apparently seems to contradict the notion of individual autonomy in decision-making. However, solidarity and communal decision-making recognize the significant role of “others” in the decision-making for clinical trial participation in certain contexts. This recognition acknowledges that respondents are embedded in communal lives, where other members of the community contribute to the health and welfare of others, and therefore could contribute to decision-making about healthcare seeking, including clinical trial participation. Communal decision-making acknowledges that individuals are also social persons who reside within a particular socio-cultural context, which influences those individuals’ viewpoints as well as their normative set of values and ideals, including those values regarding decisions for and the informed consent process for research participation. This is not ethically problematic (does not compromise voluntariness for research participation) as long as individuals remain with the option to make the final decisions regarding research participation.

What and how information is provided clearly influence decision, therefor disclosure of research-related information to potential participants influences voluntariness, even in emergency research. Improved methods of providing information may increase enrollment by reducing uninformed or underinformed refusals. Thus, using diverse forms of communicating research-related information (such as brochures, television or posters) may increase both acceptability and comprehension. However, detailed information may prolong the pre-recruitment procedures. In addition, providing information about preliminary trial results may also influence decisions to participate. This may increase ability of participants to comprehend the information, and may allow potential participants opportunity to ponder clinical research participation, to discuss their decision with family and friends, and generally to deliberate and learn more about their options. Investigators ought to employ strategies to foster, employ or strengthen culturally appropriate means of engaging potential research participants prior to or during enrollment, including solidarity and community consent. Thirdly, investigators should prioritize building trust in the healthcare system (and in research itself), by addressing research problems that address perceived community needs and through maintaining professional credibility (Additional file 1).

## Supplementary information

**Additional file 1.** In-depth research Interview guide for potential research participants (survivors of severe obstetric complications in the postnatal clinic) for the study entitled *Exploring the understanding and motivation to participate in randomized clinical trials of Emergency Obstetric and Newborn care in Uganda*.

## Data Availability

The data is available on request from the corresponding author.

## Abbreviations

RCT: Randomized Clinical trial
REC: Research and Ethics Committee
IRB: Institutional Review Board
SOMREC: School of Medicine Research and Ethics Committee

## References

[1] Notice of revised NIH definition of “clinical trial” (NIH, 23 October 2014). https://grants.nih.gov/grants/guide/notice-files/NOT-OD-15-015.html

[2] Biros MH, Lewis RJ, Olson CM, Runge JW, Cummins RO, Fost N (1995). Informed consent in emergency research. Consensus statement from the Coalition Conference of Acute Resuscitation and Critical Care Researchers. JAMA.

[3] Emanuel EJ, Wendler D, Grady C (2000). What makes clinical research ethical?. JAMA.

[4] Miller FG, Brody H (2003). A critique of clinical equipoise: therapeutic misconception in the ethics of clinical trials. Hastings Cent Rep.

[5] Shafiq N, Malhotra S (2011). Ethics in clinical research: need for assessing comprehension of informed consent form?. Contemp Clin Trials.

[6] Helmreich RJ, Hundley V, Norman A, Ighedosa J, Chow E (2007). Research in pregnant women: the challenges of informed consent. Nurs Womens Health.

[7] Vanpee D, Gillet JB, Dupuis M (2004). Clinical trials in an emergency setting: implications from the fifth version of the Declaration of Helsinki. J Emerg Med.

[8] Campbell M, Snowdon C, Francis D, Elbourne D, McDonald A, Knight R, Entwistle V, Garcia J, Roberts I, Grant A, The STEPS group (2007). Recruitment to randomised trials-strategies for trial enrolment and participation study: the STEPS study. Health Tech Assess.

[9] Carter R (2004). Application of stochastic processes to participant recruitment in clinical trials. Control Clin Trials.

[10] Gillan M, Ross S, Gilbert F, Grant A, O’Dwyer P, the Scottish Back Trial Group, the MRC Laparoscopic Hernia Group (2000). Recruitment to multicentre trials: influences. Health Bull.

[11] Halpern S (2002). Prospective preference assessment: a method to enhance the ethics and efficiency of randomized controlled trials. Control Clin Trials.

[12] Grant J (1997). Randomised trials in perinatal medicine. BJOG.

[13] Pare Toe L, Ravinetto RM, Dierickx S (2013). Could the decision of trial participation precede the informed consent process? Evidence from Burkina Faso. PLoS ONE.

[14] Rincon F, Lee K (2015). Ethical considerations in consenting critically ill patients for bedside clinical care and research. J Intensiv Care Med.

[15] Morse JM, Morse JM (1994). Emerging from the data: the cognitive processes of analysis in qualitative inquiry. Critical issues in qualitative research methods.

[16] Mays N, Pope C (2000). Qualitative research in health care: assessing quality in qualitative research. BMJ.

[17] Pope C, Ziebland S, Mays N (2000). Qualitative research in health care: analysing qualitative data. BMJ.

[18] Wong LP (2008). Data analysis in qualitative research: a brief guide to using NVivo. Malays Fam Phys.

[19] Smith JA (1996). Beyond the divide between cognition and discourse: using interpretative phenomenological analysis in health psychology. Psychol Health.

[20] Basit TN (2003). Manual or electronic? The role of coding in qualitative data analysis. Educ Res.

[21] Patton MQ (2002). Qualitative research and evaluation methods.

[22] Sutton J, Austin Z (2015). Qualitative research: data collection, analysis, and management. Can J Hosp Pharm.

[23] Larkin M, Watts S, Clifton E (2006). Giving voice and making sense in interpretative phenomenological analysis. Qual Res Psychol.

[24] Somkin C, Altschuler A, Ackerson L, Geiger A, Greene S, Mouchawar J (2005). Organizational barriers to physician participation in cancer clinical trials. Am J Man Care.

[25] Abraham N, Young J, Solomon M (2006). A systematic review of reasons for nonentry of eligible patients into surgical randomized controlled trials. Surgery.

[26] Hayden C (2007). Taking as giving: bioscience, exchange, and the politics of benefit-sharing. Soc Stud Sci.

[27] Fisher JA (2013). Expanding the frame of “voluntariness” in informed consent: structural coercion and the power of social and economic context. Kennedy Inst Ethics J.

[28] Molyneux CS, Peshu N, Marsh K (2005). Trust and informed consent: insights from community members on the Kenyan coast. Soc Sci Med.

[29] Burgess E, Singhal N, Amin H, McMillan D, Devrome H (2003). Consent for clinical research in the neonatal intensive care unit: a retrospective survey and a prospective study. Arch Dis Child Fetal Neonatal Ed.

[30] Mason S, Allmark P (2000). for the Euricon Study Group: Obtaining informed consent to neonatal randomised controlled trials: interviews with parents and clinicians in the Euricon study. Lancet.

[31] Baker L, Lavender T, Tincello D (2005). Factors that influence women's decisions about whether to participate in research: an exploratory study. Birth.

[32] Singhal N, Oberle K, Burgess E, Huber-Okrainec J (2002). Parents' perceptions of research with newborns. J Perinatol.

[33] Mohanna K, Tunna K (1999). Withholding consent to participate in clinical trials: decisions of pregnant women. BJOG.

[34] Snowdon C, Elbourne D, Garcia J (2006). It was a snap decision: parental and professional perspectives on the speed of decisions about participation in perinatal randomised controlled trials. Soc Sci Med.

[35] Rodger M, Makropoulos D, Walker M, Keely E, Karovitch A, Wells P (2003). Participation of pregnant women in clinical trials: will they participate and why?. Am J Perinatol.

[36] Zupancic J, Gillie P, Streiner D, Watts J, Schmidt B (1997). Determinants of parental authorization for involvement of newborn infants in clinical trials. Pediatrics.

[37] Chadwick R, Berg K (2001). Solidarity and equity: new ethical frameworks for genetic databases. Nat Rev Genet.

[38] Ahola-Launonen J (2016). Humanity and social responsibility, solidarity, and social rights. Camb Q Healthc Ethics.

[39] Wendler D, Shah S (2015). Involving communities in deciding what benefits they receive in multinational research. J Med Philos.

[40] Jennings B (2018). Solidarity and care as relational practices. Bioethics.

[41] Eckenwiler L (2018). Displacement and solidarity: an ethic of place-making. Bioethics.

[42] McKneally MF, Martin DK (2000). An entrustment model of consent for surgical treatment of life-threatening illness: perspective of patients requiring esophagectomy. J Thorac Cardiovasc Surg.

[43] Marsh V, Kamuya D, Rowa Y, Gikonyo C, Molyneux S (2008). Beginning community and stakeholder engagement at a busy biomedical research programme: experiences from the KEMRI CGMRC-Wellcome Trust research Programme, Kilifi. Kenya Soc Sci Med.

[44] Marsh VM, Kamuya DM, Mlamba AM, Williams TN, Molyneux SS (2010). Experiences with community and stakeholder engagement and informed consent in a genetic cohort study of severe childhood diseases in Kenya. BMC Med Ethics.

[45] Tindana P, de Vries J, Campbell M, Littler K, Seeley J, Marshall P (2015). Community and stakeholder engagement strategies for genomic studies in Africa: a review of the literature. BMC Med Ethics.

[46] Pratt B, Lwin KM, Zion D, Nosten F, Loff B, Cheah PY (2015). Exploitation and community and stakeholder engagement: can community advisory boards successfully assume a role minimising exploitation in international research?. Dev World Bioeth.

