# Supplementary material for: Lay persons’ perception of the requirements for research in emergency obstetric and newborn care
Source: BMC Med Ethics. 2021 Jan 2;22:1. doi: 10.1186/s12910-020-00568-1 (PMC7777394; doi:10.1186/s12910-020-00568-1)
Supplement: Supplementary file 1 — Additional file 1. In-depth research Interview guide for potential research participants (survivors of severe obstetric complications in the postnatal clinic) for the study entitled Exploring the understanding and motivation to participate in randomized clinical trials of Emergency Obstetric and Newborn care in Uganda. [file 12910_2020_568_MOESM1_ESM.docx]

**Exploring the understanding and motivation to participate in randomized clinical trials of Emergency Obstetric and Newborn care in Uganda**

**In-depth research Interview guide for potential research participants (survivors of severe obstetric complications in the postnatal clinic)**

**Interview characteristics**

Date of the interview: ……………………… Start time of the interview: ……………..

End time of the interview: ………………… Hours: …………………………………..

Interview number: …………………….

**Participant information**

Thank you for accepting to be interviewed. We would like to assess how much you know about research and how people make the decision to participate or not participate in research involving emergency obstetric and newborn care. The discussion shall take about 30-45 minutes. We request your permission to audio record the discussion we shall have with you. This information recorded will not mention your personal details such as names or telephone numbers. All the information that we collect will be kept confidentially and will not mention your personal details. Be free to say whatever you want to say as there is no right or wrong answer.

Please feel free to ask any questions.

**Interview questions**

1. What is your opinion about research on emergency obstetric and newborn care?

Probe for:

- 1. Is it necessary to conduct such research?
  2. How should researchers deliver information inviting participants?
  3. How should researchers try to check whether participants have understood the information?
  4. What could be the potential benefits of this research?
  5. What could be the potential risks of this research?
  6. Should there be *compensation* for adverse events or any harms that may arise during the research? Probe for: What kind of adverse events or harms could potentially occur and what form of compensation could be availed to participants

1. What factors may affect how participants understand the given information?

Probe for:

- 1. Nature of disease
  2. Severity of disease?
  3. Language?
  4. The way information is presented?
  5. Timing of the information?
  6. Ongoing medication?
  7. Complexity of consent document?
  8. The contextual factors such as research environment, privacy, confidentiality?

1. In your view, what do you think are some of the reasons or factors that influence decisions to participate in research in emergency contexts?

Probe for:

- 1. What could be incentives?
  2. Is it financial?
  3. Is there a need to promote scientific advancement?
  4. Contextual factors?
  5. Medical benefit?
  6. Family and friends?
  7. Healthcare providers and relationship with them?

**Thank you very much for your participation**
